# Optogenetic control of NOTCH1 signaling

**DOI:** 10.1186/s12964-022-00885-5

**Published:** 2022-05-18

**Authors:** Joanna Kałafut, Jakub Czapiński, Alicja Przybyszewska-Podstawka, Arkadiusz Czerwonka, Adrian Odrzywolski, Cecilia Sahlgren, Adolfo Rivero-Müller

**Affiliations:** 1 Department of Biochemistry and Molecular Biology, Medical University of Lublin, 21-093 Lublin, Poland; 2 Faculty of Science and Engineering, Biosciences, Åbo Akademi, Turku, Finland; 3 Institute for Complex Molecular Systems, Eindhoven University of Technology, Eindhoven, The Netherlands

**Keywords:** Optogenetics, Notch signaling, NOTCH1, Light-activation, Breast cancer

## Abstract

The Notch signaling pathway is a crucial regulator of cell differentiation as well as tissue organization, whose deregulation is linked to the pathogenesis of different diseases. NOTCH1 plays a key role in breast cancer progression by increasing proliferation, maintenance of cancer stem cells, and impairment of cell death. NOTCH1 is a mechanosensitive receptor, where mechanical force is required to activate the proteolytic cleavage and release of the Notch intracellular domain (NICD). We circumvent this limitation by regulating Notch activity by light. To achieve this, we have engineered an optogenetic NOTCH1 receptor (optoNotch) to control the activation of NOTCH1 intracellular domain (N1ICD) and its downstream transcriptional activities. Using optoNotch we confirm that NOTCH1 activation increases cell proliferation in MCF7 and MDA-MB-468 breast cancer cells in 2D and spheroid 3D cultures, although causing distinct cell-type specific migratory phenotypes. Additionally, optoNotch activation induced chemoresistance on the same cell lines. OptoNotch allows the fine-tuning, ligand-independent, regulation of N1ICD activity and thus a better understanding of the spatiotemporal complexity of Notch signaling.

**Video Abstract**

**Supplementary Information:**

The online version contains supplementary material available at 10.1186/s12964-022-00885-5.

## Background

Notch signaling pathway is an evolutionarily conserved cell communication system present in most multicellular organisms. This pathway plays essential roles during cell fate determination and thus during development and tissue homeostasis. Generally, the Notch system controls lateral inhibition and lateral induction, binary cell fate, and boundary formation during embryogenesis [1]. Consequently, Notch signaling plays key roles in vasculature formation [2], osteogenesis [3, 4], and plasticity of the nervous systems [5, 6]. Cell-to-cell contact between a cell expressing a NOTCH receptor and a ligand-expressing cell is the basis for activation of Notch signaling. NOTCH transmembrane receptors (NOTCH1-4) are formed of a large extracellular domain (ECD), a transmembrane domain (TMD), and an intracellular domain (NICD). The ligands, Delta/Serrate/Lag2 family of proteins also have long ECDs followed by a TMD and an ICD [7, 8]. The ECD of every NOTCH receptor is cleaved, by a furin-like convertase, at Site-1 (S1) during biosynthesis but remains non-covalently attached to the rest of the receptor, allowing for detachment from the rest of the receptor upon mechanical pulling by any of the ligands. In turn, the unfolding created by the mechanical removal of the large ECD uncovers a cryptic site that is then recognized by proteases (ADAM10, ADAM17 [9], and later on gamma-secretase [10]) causing a series of proteolytic events (S2, S3 and S4) leading to the release of NICD from the membrane. The NICD then translocates to the nucleus where it converts the transcriptional repressor CSL (also known as RBP-J) into a transcriptional activator of Notch-targeted genes [11], such as *HEY* (Hairy/enhancer-of-split related with YRPW motif), *HES* (Hairy/Enhancer of split [E(spl)]) families of genes, as well as *MYC* (c-Myc protein), *DTX* (Deltex E3 ubiquitin ligase) and *NRARP* (NOTCH regulated ankyrin repeat protein) [12].

Besides physiological processes, Notch signaling plays roles in progression, migration, invasion, and metastasis in several human malignancies. Additionally, its upregulation is often associated with poor prognosis and drug resistance [13, 14]. In breast cancer (BC), Notch signaling is related to the control of autophagy, apoptosis [15], cancer cell stemness, and chemosensitivity [16]. Moreover, NOTCH and, in particular, its ligand JAG1 have also been implicated in cluster-cell migration and metastasis of BC [17]. Thus, the monitoring of Notch-related gene expression patterns, as well as control of Notch signaling, is likely to have therapeutic potential in BC [18–21].

Notch signaling is pleiotropic and depending on the ligand type presented by the sending cell, as well as the endogenous context of the receiving cell (with receptor), the outcome might be different [22–24]. This is allegedly the result of different modes of activation e.g. short impulse vs long activation patterns [25], as the result of the ligands affinity for the ECD of NOTCH receptors [26, 27], further affected by other cellular signaling events [23, 24]. Therefore, to effectively study Notch signaling, proper tools that allow temporal control of Notch activation are needed.

Optogenetics utilizes light-sensitive proteins to stimulate biological processes in an illumination-dependent manner. Optogenetic tools enable non-invasive, flexible, and inexpensive modulation of biological processes such as activation or inactivation of biological pathways [28, 29], control of gene expression [30, 31], and DNA recombination [32]. Photo-activation has many advantages compared to traditional methods, such as the use of chemicals or genetic systems. Light control avoids off-target interactions between chemicals and cellular components. Additionally, light can be precisely directed to a single cell, or an area of a cell, and can be carefully controlled in intensity and duration.

Here, we show that an engineered OptoNotch (oN) system can regulate NOTCH1 activity with spatiotemporal precision. We apply oN to induce BC chemoresistance, cell proliferation and spheroids growth. The fine-tuned regulation of NOTCH1 activity makes oN an excellent tool to study the role of Notch signaling in embryogenesis, cancer biology, and drug resistance.

## Materials and methods

### Materials

KOD-Xtreme hot-start DNA polymerase (Merck Millipore), DreamTaq™ Green PCR Master Mix (ThermoFisher Scientific), *DpnI* restriction enzyme (Thermo Fisher Scientific), Gibson Assembly^®^ Master Mix (NEB), Ampicillin (BRAND), Kanamycin (Sigma Aldrich), Spectinomycin (Sigma Aldrich), Doxorubicin (Sigma Aldrich), Paclitaxel (Sigma Aldrich). DNA Clean & Concentrator and Zyppy Plasmid Kits (Zymoresearch), Turbofect™ Transfection Reagent (ThermoFisher Scientific), Lipofectamine^®^ 3000 Reagent (Invitrogen), Bright-Glo Luciferase Assay System (Promega), Vibrant DiD Cell-Labeling Solution (ThermoFisher Scientific), DAPT (Sigma Aldrich), Dulbecco’s Modified Eagle Medium (DMEM/F-12), Penicillin and Streptomycin (Sigma Aldrich), fetal bovine serum (PromoCell) and geneticin G418 (ThermoFisher Scientific). Plasmids pTriEx-NTOM20-LOV2 and pTriEX-mCherry-Zdk1 were a gift from Klaus Hahn (Addgene plasmid # 81009; http://n2t.net/addgene:81009; RRID:Addgene_81009 and Addgene plasmid # 81057; http://n2t.net/addgene:81057; RRID:Addgene_81057) [33]. Plasmid pDONR223_NOTCH1_ICN was a gift from Jesse Boehm & William Hahn & David Root (Addgene plasmid # 82,087; http://n2t.net/addgene:82087; RRID:Addgene_82087) [34]. NOTCH1 activity reporters 12xCSL-Luc have previously been described [35]. Cell lines HEK293T, MDA-MB-468, and MCF7 were obtained from ATCC. Primary anti-NOTCH1 (D1E11) rabbit monoclonal antibody (Cell Signaling Technology, Cat. Nr: #3608), secondary anti-rabbit antibody conjugated with AlexaFluor 532 (Invitrogen Cat. Nr: #A-11009). Secondary antibody: Peroxidase F(ab’)_2_ Fragment Donkey Anti-Rabbit IgG (H + L) (711-036-152, Jackson ImmunoResearch). Immunocytochemistry reagents: Alexa Fluor 488 Tyramide Reagent (B40953, LifeTechnologies), Hoechst 33342 (Cayman), ProLong Gold mounting medium (LifeTechnologies). All PCR primers were bought from Genomed (Warsaw, Poland).

### Molecular cloning

The construct MTS-LOV2-P2A-Zdk1-N1ICD (full sequences in Additional file 1) was generated by Gibson Assembly by combining products from PCR reaction. The Light-oxygen-voltage-sensing domain 2 (LOV2) sequence was amplified from pTriEx-NTOM20-LOV plasmid, Zdark 1 (Zdk1) from pTriEX-mCherry-Zdk1, and human NOTCH1 intracellular domain (N1ICD), lacking the S4 and S3 cleavage sites, from pDONR223_NOTCH1_ICN. All PCR reactions were performed using KOD-Xtreme high-fidelity polymerase according to the manufacturer’s protocol. Next, PCR products were digested with *DpnI* restriction endonuclease to remove methylated template plasmids and purified using DNA purification kits. The Gibson Assembly reaction was performed according to the original protocol [36]. Next, the reaction product was transformed into previously prepared electrocompetent *E.coli* bacteria. The resulting bacterial colonies, after selection with kanamycin 50 µg/mL, were subjected to colony PCR and sequence-verified.

Next, the MTS-LOV2-P2A-Zdk1-N1ICD plasmid was used for creating LOV2 mutation (LOV2^V416L^). The sequences of the primers for LOV2^V416L^ in Additional file 1: Table S1.

### Cell culture and transfection

Human embryonic kidney 293T (HEK293T) cells and breast cancer cell lines (MDA-MB-468 and MCF7) were cultured in Dulbecco’s Modified Eagle Medium (DMEM/F-12). All cells were supplemented with 10% FBS, penicillin (100 units/mL)/streptomycin (100 μg/mL) and kept in an incubator at 37 °C in an atmosphere of 5% CO_2_.

One day before transfection, HEK293T cells were seeded 5 × 10^4^ cells/well into a 24-well plate and co-transfected MTS-LOV2^V416L^-P2A-Zdk1-N1ICD and the Notch reporter 12xCSL-Luc plasmid using Turbofect™ transfection reagent following the manufacturer’s protocol. Mock transfection contains GFP as a transfection control, and 12×CSL-Luc for measuring endogenous NICD levels was used as a negative control (Nc). All experiments were done in, at least, triplicate.

Similarly, one day before transfection, MCF7 and MDA-MB-468 cells were seeded 5 × 10^4^ cells/well into a 24-well plate. Medium with 2% FBS was used 24 h before MCF7 transfection to increase transfection efficiency. For transfection with MTS-LOV2^V416L^-P2A-Zdk1-N1ICD Lipofectamine^®^ 3000 was used, following the manufacturer’s protocol. After 48 h the cells were selected with geneticin (G418) (0.75 mg/ml) for over three weeks to generate stable lines.

### Immunostaining and flow cytometry

Detection expression of MTS-LOV2^V416L^-P2A-Zdk1-N1ICD construct (optoNotch, oN) into MCF7 and MDA-MB-468 stable cell lines were assessed by measurement of the fluorescent intensity from binding the primary anti-NOTCH1 (D1E11) rabbit monoclonal antibody and secondary anti-rabbit antibody conjugated with AlexaFluor532. Cells were fixed by resuspending in fixation and permeabilization buffer (BD Pharmingen, Cytofix/Cytoperm solution, cat. # 554722) and incubated for 20 min on ice. Next, cells were washed (BD Perm/Wash buffer, cat. numb. 554723) and centrifuged (500*xg*, 5 min). Both, WT and oN, MCF7 and MDA-MB-468 cells were incubated with primary anti-NOTCH1 antibody (1 h, 37 °C and 5% CO_2_) and subsequently after a washing step, labelled with secondary AlexaFluor532-conjugated antibody (1 h, 37 °C, and 5% CO_2_). Part of the cells was incubated only with AlexaFluor532 conjugated antibody.

All immunostainings were performed immediately before the flow cytometry analysis. For Flow Cytometry was performed using a BD FACSCalibur (BD) with CellQuest Pro Version 6.0. software. The fluorescence AlexaFluor532 intensity of individual cells was determined as Counts/FL2-H 2D-dot plots at least 10,000 events were measured within an acquisition rate of 300 events/second, approximately.

### Immunocytochemistry and confocal microscopy

oN MDA-MB-468 cells were seeded on glass bottom Labtec 8-chamber slides (Nunc) at a density of 4 × 10^5^ cells/mL. The cells on one slide were photo-activated by 5 ms blue light pulses, while those on the second slide were kept in the dark. Cells were then washed with PBS and fixed 1:1 Acetone: Methanol for 30 min at − 20 °C. Next, cells were washed with PBS and incubated in Blocking Buffer (BB) for 1 h at room temperature, followed by overnight (4 °C) incubation with primary rabbit antibodies against N1ICD diluted 1:500 in BB. After triple washes in PBS, cells were incubated for 1 h (room temperature) with Secondary Peroxidase F(ab’)_2_ Fragment Donkey Anti-Rabbit IgG (H + L) diluted 1:1000 in BB. After three PBS washes, cells were stained with Alexa Fluor 488 Tyramide Reagent following to manufacturer’s protocol. Reaction was stopped through incubation in 3% hydrogen peroxide. For additional staining of nuclei-cells were washed in PBS with Hoechst 33,342 in concentration 10 µg/mL. Next, cells were mounted with ProLong Gold mounting medium and visualized under a Nikon ECLIPSE Ti confocal microscope. Analyzes were carried out with the use of Automated Morphometric Image Data Analysis (AMIDA) software [37].

### Photoactivation

**Fig. 1:**
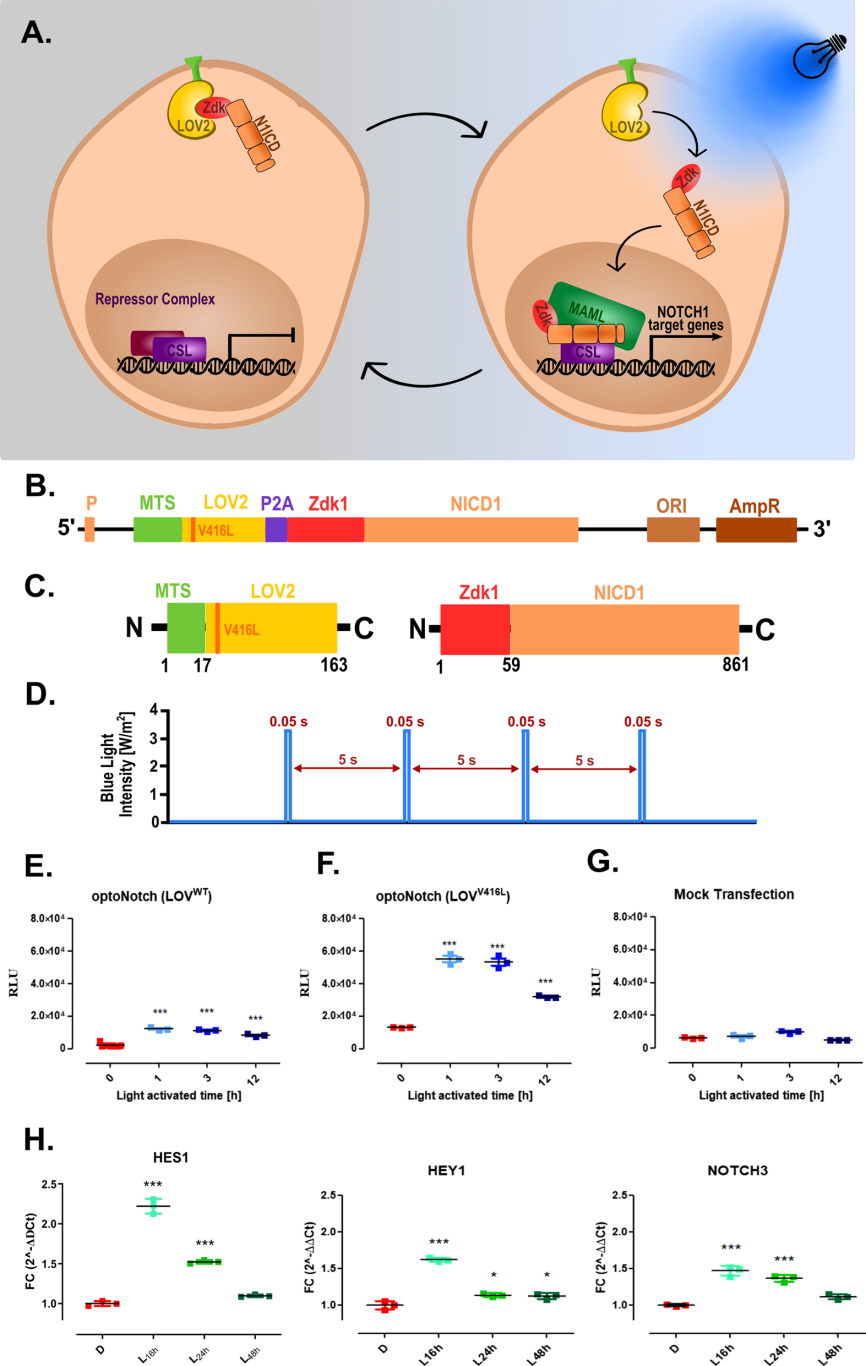
Engineering of photo-activatable Notch. Scheme of the engineered system and optogenetic model of action used throughout this study. **a** The LOV2^WT^:Zdk1-N1ICD or LOV2^V416L^:Zdk1-N1ICD is anchored in the cell membrane until photo-activation (456 nm, blue light bulb) induces dissociation of the complex, where Zdk1-N1ICD translocates to the nucleus to activate Notch-target genes. **b** The vector is designed to express LOV2^WT^ or LOV2^V416L^ mutant and Zdk1-N1ICD linked by a P2A “self-cleaving” sequence; **c** resulting proteins containing LOV2^WT^/^V416L^ (with membrane targeting sequence, MTS) and Zdk1-N1ICD. P—CMV promoter, MTS—myristoylation-targeting sequence, LOV2—light-oxygen-voltage-sensing domain 2, P2A—porcine teschovirus-1 2A, Zdk1- Zdark 1, N1ICD—human NOTCH1 intracellular domain. **d** Schematic representation of blue light activation pattern (pulses) used in optoNotch system. **e**–**g** The HEK293T cells expressing either LOV2^WT^-Zdk1-N1ICD or LOV2^V416L^-Zdk1-N1ICD were transfected by the *12xCSL*-Luc reporter and subsequently light activated. The relative luminescence units (RLU) level was measured in LOV2^WT^ (**e**) or LOV2^V416L^-Zdk1-N1ICD (**f**) and mock-transfected cells (**g**) 48 h after activation. The duration of photoactivation of optoNotch (0 h, 1 h, 3 h, and 12 h) does not equate to the increased response. **h** The mRNA expression of human *HES1*, *HEY1,* and *NOTCH3* genes was determined by qPCR (2^–∆∆Ct^) 16, 24, and 48 h after light stimulation of optoNotch (LOV2^V416L^)-HEK293T cells. The results are presented as fold change (FC) values (mean ± SD) normalized to the human *GAPDH* gene expression. All data were analyzed with a one-way ANOVA test and Tukey’s multiple comparisons post-hoc test vs. not light stimulated cells (the time point 0 h and D; dark, respectively). *p ≤ 0.05; **p ≤ 0.01; ***p ≤ 0.001 were considered statistically significant, ns – non significant

Transfected HEK293T or stably transfected oN breast cancer MCF7 and MDA-MB-468 cells were photo-stimulated using the MAGI-01 Opto-stimulation system (Radiometech) with blue LEDs (456 ± 2 nm), at an intensity of 3.2 W/m^2^, in pulses of 0.05 s luminous and 5 s breaks (Fig. 1d) for 1, 3, or 12 h, while unstimulated cells were kept in the dark. Light-stimulation was performed inside the cell culture incubator to avoid changes in environmental conditions for the photo-stimulated cells. The MAGI-01 machine and the illumination (LED) platform can be seen in the Additional file 1: Fig. S1.

### Luciferase reporter assay

At 48 h after blue light activation, oN transfected HEK293T cells were lysed following to manufacturer’s protocol. The equal volume of lysates and Bright-Glo Luciferase reagent was transferred to a black microplate well and measured using a microplate luminometer (Tecan Infinite 200 PRO). The results were then statistically analyzed.

### Proliferation assay

Either oN MCF7 or oN MDA-MB-468 cells were seeded into two 96-well plates at a density of 3 × 10^4^ cells/mL. For the next two days, one plate was activated blue light pulses by 3 h per day, while the second plate remained in the dark. 96 h after the last activation, the cells were exposed to 10µL per well of MTT solution (5 mg/mL in PBS with ions) for 3 h. After incubation, 100µL per well SDS buffer (10% SDS in 0.01 N HCl) was added to dissolve the crystals. Next, the color product of the reaction was quantified by measuring absorbance at a 570 nm wavelength using a microplate reader (Tecan Infinite 200 PRO).

### Wound-healing assay

oN MCF7 and oN MDA-MB-468 cells were labelled with Vibrant DiD and seeded into 12-well plates at a density 5 × 10^4^ cells/mL. One day later, the monolayer of cells was scratched to create a linear wound. To inhibit the endogenous Notch signaling, 10 µM DAPT was used as an additional control. Cells were activated by blue light pulses by 3 h as described above, while the second plate remained in the dark. 24 h later, the Wound-healing Assay images were performed under an EVOS M5000 Image System (ThermoFisher).

### Drug resistance assay

Both, oN MCF7 and oN MDA-MB-468 cells, were independently seeded at a density of 4 × 10^4^ cells/mL into two 96-well plates. The next day, the medium was removed and replace with fresh complete growth media with different concentrations of drugs. Doxorubicin was used at concentrations of 0.1; 0.25; 0.5; 1, 2, 3, 4, 5 and 10 µM. Paclitaxel was used at the following concentrations: 1; 2.5; 5; 7.5; 10; 15; 20; 25 and 50 nM. For the next three days, one of the twin plates was activated by blue light pulses for 3 h per day, while the second remained in the dark. On the fourth day, the cells were exposed to 10 µL per well of MTT solution (5 mg/mL in PBS with ions) and incubate for 3 h. After incubation, 100µL per well SDS buffer was added. The color product was quantified by measuring absorbance at 570 nm using a microplate reader.

### Cell counting

To determine the number of cells, oN MCF7 and oN MDA-MB-468 were seeded into two 24-well plates at a density of 5 × 10^4^ cells/mL. For the next two days, one plate was activated blue light pulses by 3 h per day, while the second plate remained in the dark. 96 h after the last activation, the cells were counted using a TC20™ Automated Cell Counter (Bio-Rad). All measurements were made in triplicate.

### 3D spheroid cultures

Both MCF7 and MDA-MB-468, wild type (WT) controls or oN, were seeded 50 µL per well into two 96 Well Round U-Bottom Plates, Sphera Low-Attachment Surface (Thermo Fisher) at a density of 4 × 10^4^ cells/mL. The next day into wells with spheroids was added Matrigel (Corning) dissolved in medium with the addition of 10 µM of DAPT to inhibit endogenous NOTCH signaling. The plates were allowed to polymerize overnight at 37 °C. One plate was blue light (456 nm) activated with an intensity of 3.2 W/m^2^ in pulses of 0.05 s every 5 s for 3 h every day during the experiment, while the second plate has remained in the dark. The spheroids area was monitored for 8 days. All spheroids were grown in 7 replicates.

### Microscopy of living cells

Images and area measurements were obtained using an EVOS M5000 Imaging System. The photos were taken on the day Matrigel was added (time 0) and then every two days (2, 4, 6, and 8 days). Each spheroid was visualized daily using identical microscope settings.

### 3D sandwich assay

The Matrigel at a concentration of 4 mg/ml in medium (Lower gel) was poured onto the bottom of a 96-well plate, and incubated at 37 °C for 30–60 min. Then, single cells suspension of either, MCF7 or MDA-MB-468, WT or oN, were admixed with Matrigel (Upper gel, final concentration of 2 mg/ml of Matrigel) and seeded at a density of 4 × 10^4^ cells/mL. The gamma-secretase inhibitor (DAPT) was added into the upper gel at a concentration of 10 µM. The plates were centrifuged for 10 min, and incubated at 37°C overnight. The cells in one plate were light activated by pulses of 0.05 s every 5 s for 3 h every day during the experiment, while an identical second plate remained in the same conditions but in the dark. The spheroids area was visualized under Nikon Ti Confocal microscope on day 0, 3, 6, 9 and 12.

### RNA extraction and RT-qPCR

Trypsin was used to detach the cells after the experiment. Cell were then collected by centrifugation (400×*g*). After removing the supernatant, the cells pellets were lysed according to the ExtractMe Total RNA kit (Blirt) manufacturer’s protocol. cDNAs were synthesized using the High-Capacity cDNA Reverse Transcription Kit with the addition of a RNase Inhibitor (Applied Biosystems). All primers used for qPCR were tested for specificity and sensitivity. Glyceraldehyde 3-phosphate dehydrogenase (*GAPDH*) was used as a housekeeping gene. PCR reactions were performed with PowerUp SYBR Green Master Mix (Applied Biosystems) through the LightCycler^®^ 480 II instrument (Roche) in triplicates on 96-well plates. The number of cycles needed to reach a specific threshold of detection (CT) was used to calculate relative quantification (RQ). Relative mRNA expression was calculated using the delta CT subtraction and normalized to the expression of *GAPDH*.

### Statistical analyses

Significance among luminescence readings was assessed by one-way ANOVA followed by Tukey’s *post-hoc* test. Statistical analyses of all samples were performed using GraphPad Prism 8.0 (GraphPad Software Inc., California, U.S.A). ANOVA with Tukey post hoc test and column statistics were used for comparisons (*p ≤ 0.05; **p ≤ 0.01; ***p ≤ 0.001 was considered statistically significant). All tests were performed in the triplicates, at least.

## Results and discussion

NOTCH1 was re-engineered so the N1ICD is released from a tethered membrane location upon blue light excitation. To achieve that we adapted the reversible optogenetic system LOVTRAP. This system is based on two proteins, Light-oxygen-voltage-sensing domain 2 (LOV2) and Zdark 1 (Zdk1), that dimerize in the dark but dissociate under blue light stimulation [33]. We anchored LOV2 to the cell membrane, the natural location of inactive NICD, via the Myristoylation-targeting sequence (MTS), while Zdk1 was fused to the intracellular domain of the human NOTCH1 (N1ICD), lacking the S3 to avoid influence by gamma-secretase (Fig. 1a). To facilitate the expression of both proteins in cells, we joined them through a P2A (porcine teschovirus-1 2A) “self-cleaving” sequence so that both could be expressed within one reading frame (Fig. 1b). The P2A is one with a self-cleaving peptide, which enables the formation of two separate proteins during translation (Fig. 1c). In the dark, LOV2 and Zdk1-NICD shall localize at the cell membrane. Upon release from the cell membrane, the N1ICD translocates to the nucleus where it acts as a transcriptional activator together with CSL and MAML (Fig. 1a). To measure N1ICD activity, we employed a well-known reporter system where the activity of N1ICD correlates to the downstream expression of Firefly Luciferase (*12xCSL-Luc*) [35].

We initially engineered a construct containing wild-type (WT) LOV2, however, the dissociation of Zdk1 from LOV2 is a reversible reaction, and they rapidly reunite in the absence of illumination with high affinity. This reaction resulted in a very low reporter (luciferase) signal after photoactivation of the optoNotch system no matter the length of activation (0.05-s pulses for 1, 3, or 12 h) (Fig. 1e). We then generated the V416L LOV2 variant, which is known to result in a slower regain of affinity for Zdk1 after photo-dissociation [33]. This mutant (LOV2^V416L^) showed a clear increase in reporter expression upon blue light activation (0.05-s pulses for 1, 3, and 12 h) by approximately fivefold as compared to the WT LOV2 (LOV2^WT^) (Fig. 1f).

We next evaluated the effects of short or longer light activation on downstream reporter activity. We expected that the increased length of photoactivation will also result in higher reporter activity. To our surprise, that was not the case, 1 and 3 h activation resulted in identical reporter values while 12 h light exposure resulted in a reduced luciferase signal (Fig. 1f). Control experiments on mock transfected cells, having the reporter system, under identical conditions showed that blue light has no effect on reporter expression (Fig. 1g).

To confirm that optoNotch (oN) retains the gene targeting functions of endogenous N1ICD, we analyzed the response of Notch-target genes by qPCR after illumination. For this purpose, *HES1*, *HEY1,* and *NOTCH3* genes were selected, whose expression is known to be dependent on NOTCH1 activity [38, 39]. We photoactivated oN-expressing cells for 3 h and analyzed the responsive genes 16, 24, and 48 h after. This experiment had 2 purposes: first, to determine whether optoNotch still targets the same genes as the native N1ICD, and second that its activation is reversible without further photo-activation. All three target genes showed increased expression in light-activated cells (Fig. 1, H, L samples) as compared to the same cells kept in the dark (Fig. 1, D samples). The greatest increase of target gene expression was observed 16 h after light activation. As expected, without further photoactivation of optoNotch the expression of target genes slowly returned to their original levels in about 48 h (Fig. 1f)﻿.

### OptoNotch on breast cancer cell proliferation

**Fig. 2:**
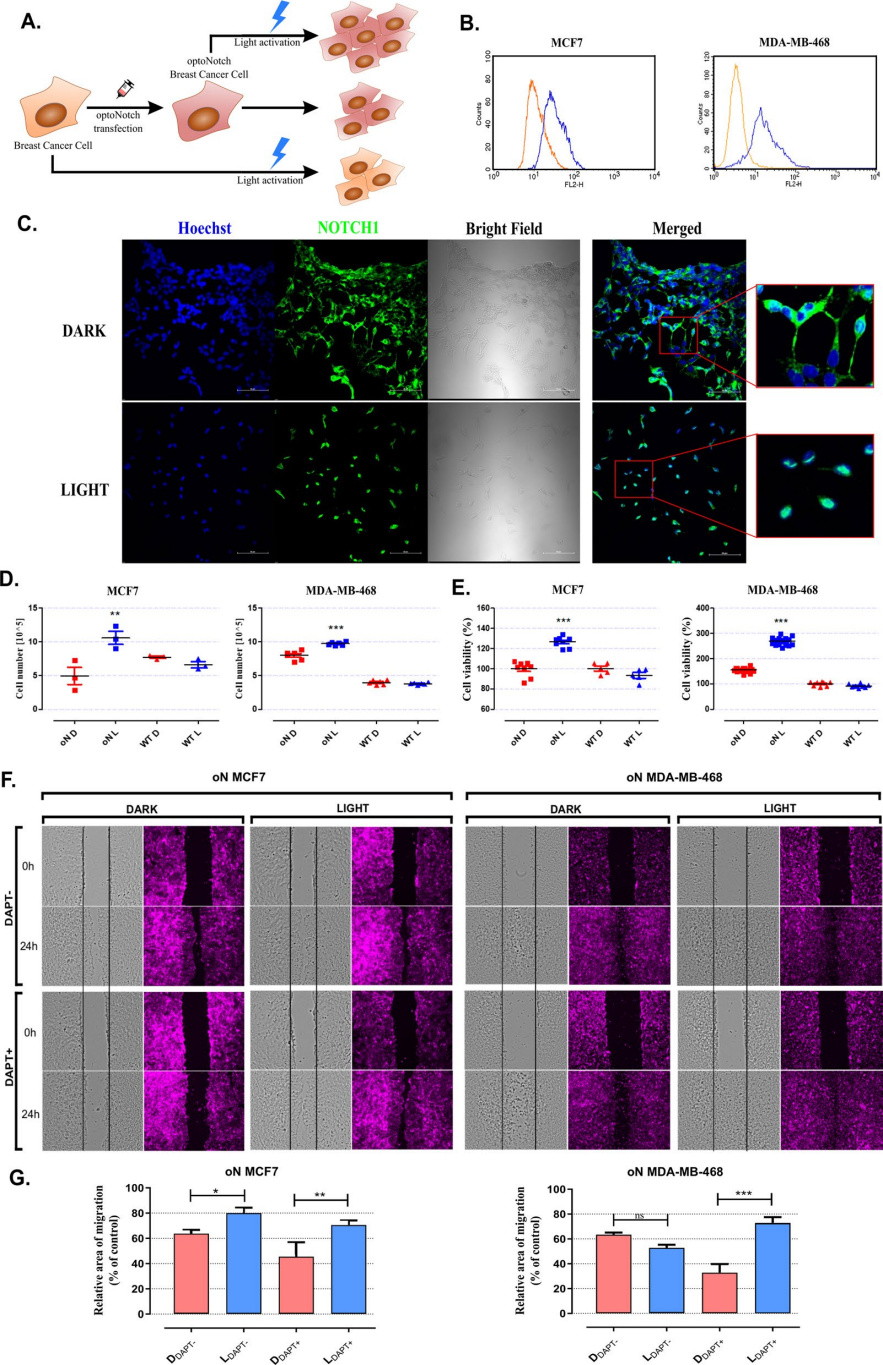
Functional effects of light activation of optoNotch (oN) stably expressing breast cancer cell lines. Schematic representation of the oN on cell proliferation (**a**). The level of N1ICD was determined by immunostaining using flow cytometry in WT (orange) and LOV2^V416L^-Zdk1-N1ICD stable expressing (blue) MCF7 and MDA-MB-468 (**b**) cell lines. Immunocytochemical staining of N1ICD (green) in oN MDA-MB-468 cells (**c**). For better visualization, Hoechst 33,342 was used for nuclei (blue) staining. Number of N1ICD stable expressing BC cells (**d**). Cells were activated by 0.05 s blue light pulses for 3 h per day. After 96 h, MCF7 and MDA-MB-468 cells were counted in a TC20™ Automated Cell Counter. The results (mean ± SD) show cell number values. Simultaneously, MCF7 and MDA-MB-468 relative cell viability was measured by the MTT assay (**e**). The results (mean ± SD) show relative cell viability (% of control—WT D) of MCF7 and MDA-MB-468 cell lines, respectively. Influence of Notch signaling inhibitor (DAPT) on oN MCF7 and oN MDA-MB-468 cells unstimulated (DARK) or photo-stimulated (LIGHT) assessed by Wound-healing Assay. Representative images of the cells migration (**f**) and graphs of relative area of cells migration into wound area (percent of control) (**g**) were shown.. oN D—LOV2^V416L^-Zdk1-N1ICD stably expressing cells kept in the dark; oN L—light-activated LOV2^V416L^-Zdk1-N1ICD stably expressing cells; WT D—wild-type cells kept in the dark; WT L – light-activated WT cells. All data were analyzed with a one-way ANOVA test and Tukey’s multiple comparisons post-hoc test vs. not light stimulated cells (D; dark). *p ≤ 0.05; **p ≤ 0.01; ***p ≤ 0.001 were considered statistically significant

Since NOTCH1 signaling is a well-known player in BC development [40], we selected two BC cell lines, the triple-negative (TNBC) MDA-MB-468 and the estrogen-positive MCF7 cells, to assess the functionality of optoNotch. Due to the low efficiency of transient transfection in these cell lines, we generated stable lines expressing the MTS-LOV2^V416L^-P2A-Zdk1-N1ICD complex (oN) (Fig. 2a)—from here onwards referred to as oN MCF7 and oN MDA-MB-468, respectively. To ensure the expression oN, we analyzed these cells by immunostaining the N1ICD using flow cytometry, where the total content of N1ICD in both of these stable cell lines was substantially higher than in WT cells (where there is some endogenous N1ICD) (Fig. 2b).

Immunofluorescent labelling was performed to confirm the cellular localization of the N1ICD before and after light activation. For this purpose, oN MDA-MB-468 cells were used. As expected, N1ICD resides mainly at the cell membrane prior to activation. Photo-activation leads to the dissociation of Zdk1-N1ICD from the membrane tethered LOV2, and relocation into the cell nucleus (Fig. 2c).

To confirm that Zdk1-N1ICD retains the ability to activate Notch-dependent genes in BC cells, the expression of *HES1*, *HEY1* and *NOTCH3* mRNA was analyzed before and after light activation. The results were normalized to the expression level of the same genes in WT BC cells. In both, oN MCF7 and oN MDA-MB-468 cells, the expression of *HES1* was a statistically significant higher after blue light activation (Additional file 1: Fig. S2). Interestingly, the response of *HEY1* and *NOTCH3* genes differed between cell lines. A statistically significant increase in *HEY1* expression was characteristic for MDA-MB-468, while an increase in *NOTCH3* expression was observed in MCF7 (Additional file 1: Fig. S2).

We then proceeded to determine the rate of cell growth with and without light activation in oN and WT BC cells. The cells were activated by blue light in 0.5 s pulses for 1 h per day, as this time was the setting resulting in highest reporter gene expression (Fig. 1E). After 96 h, cell number was determined by an automated cell counter. In a parallel experiment, relative cell viability (calculated as the quantity of living cells at the end of the experiment) was determined by the MTT assay. Both oN-MCF7 and oN-MDA-MB-468 cells in each test showed an increased number of cells after light-activation (oN L) as compared to the same cells kept in the dark (oN D). While the WT cells of both lines, whether light-activated (WT L) or not (WT D), maintained an identical relative cell viability rate between them (Fig. 2d, e).

The wound-healing assay was performed to test the effect of oN on proliferation and migration of BC cells. To block the activity of endogenous Notch, gamma-secretase inhibitor (GSI) DAPT was added to the cell cultures. Unstimulated oN MCF7 cells cultured without DAPT, migrated 20% less than photoactivated oN cells after 24 h (Fig. 2f, g). The highly migrating oN MDA-MB-468 cells, photo-stimulated or not, migrated equally fast in the absence of DAPT (Fig. 2f, g). Addition of DAPT successfully inhibited cell migration of both unstimulated cell lines, yet such inhibition was fully rescued by light activation of oN in both cell lines (Fig. 2f lower panels). The fact that light activation of both oN cells with DAPT fully compensates cell migration (Fig. 2g), further supports the role of Notch1 signaling in this process.

**Fig. 3:**
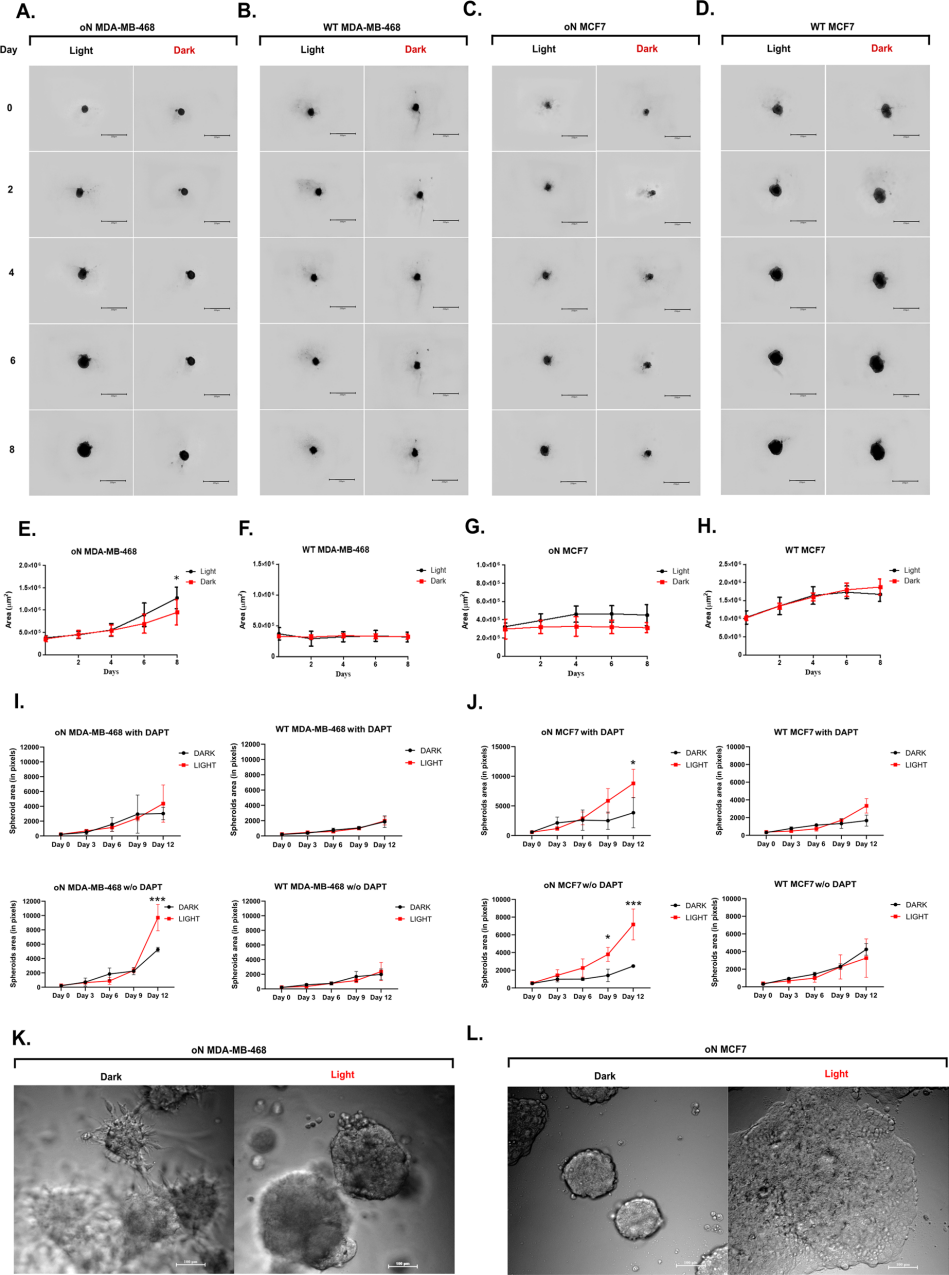
The total size of breast cancer cell spheroids. The LOV2^V416L^-Zdk1-N1ICD (oN) and wild type (WT) MCF7 and MDA-MB-468 cells were cultivated in Matrigel with 10 µM DAPT in duplicated 96-well plates. **a** MDA-MB-468 and **c** MCF7 cells expressing LOV2^V416L^-Zdk1-N1ICD (oN) and wild type (WT) cells (**b**, **d**, respectively) are shown. Light (red line)—cells light-activated for 3 h every day; Dark (black lines)—not light stimulated cells. **e**–**h** representative graphs of stable LOV2^V416L^-Zdk1-N1ICD and WT breast cancer spheroids were taken every 48 h for the duration of the experiment (8 days). The spheroids (n = 7) area was analyzed every 48 h for 8 days. Area of spheroids MDA-MB-468 (**i**) and MCF7 (**j**), oN and WT, obtained through sandwich assay, which is characterized by the growth of spheroids from single cells. Spheroids were cultured between two layers of Matrigel (Lower gel − 4 mg/ml and Upper gel − 2 mg/ml) with 10 µM DAPT and controls without this inhibitor. Each spheroid area was analyzed every 72 h for 12 days through AMIDA software. Light (red line)—cells light-activated for 3 h every day; Dark (black lines)—not light stimulated cells. Comparison of the morphology of spheroids formed by oN MDA-MB-468 (**k**) and oN MCF7 (**l**) photo-stimulated (LIGHT) or kept in the dark (DARK)

To confirm the influence of the optoNotch system on BC development under physiological-mimicking conditions, both lines with and without oN were cultured in 3D spheroids using Matrigel. DAPT (10 µM) was used to rule out an effect on the growth of spheroids by endogenous Notch activity. Photo-activation of oN induced increased proliferation—the size of the spheroids increased faster—than those non-illuminated (Fig. 3a, c, e, g), or WT cells whether illuminated or not (Fig. 3b, d, f, h).

The sandwich assay was used to confirm the results from single-cell-derived spheroids. To exclude the influence of endogenous Notch, the experiments were performed in the presence, or absence in control samples, of DAPT. Analyzes were carried out with the use of Automated Morphometric Image Data Analysis (AMIDA) software [37], which allowed the quantitative measurements of the size of at least 75 spheroids per sample. In the case of oN MDA-MB-468 cells, in the presence of DAPT, light activation caused a noticeably increased growth of spheroids by day 12. WT MDA-MB-468 cells showed equal growth rate, with DAPT affecting them equally, whether photo-stimulated or not (Fig. 3i). Similarly, light stimulated oN MCF7 cells showed a significant increase in spheroid growth, irrespective of the presence of DAPT (Fig. 3j), representative images are presented in Additonal file 1: Fig S3﻿. Moreover, photo-activation of oN cells, not only increased cell proliferation rate, but also have an influence on migration and spheroid morphology. The non-activated oN MDA-MB-468 cells showed a greater tendency for single-cell migration. However, activating oN accelerated proliferation but seemed to make cells less likely to migrate out of the spheroids (Fig. 3k). The exact opposite trend was observed in oN MCF7 cells. Where activation of the oN system not only led to a higher rate of proliferation, but also increased the tendency of cells to migrate (Fig. 3l). This somehow surprising finding points out to the pleiotropic roles of Notch signaling in different cell types. This finding also correlates with the scratch assay, where oN MCF7 cells grow and migrate more upon light-activation, while oN MDA-MB-468 cells grow more but with seemed reduced migration (Fig. 2f, g).

**Fig. 4:**
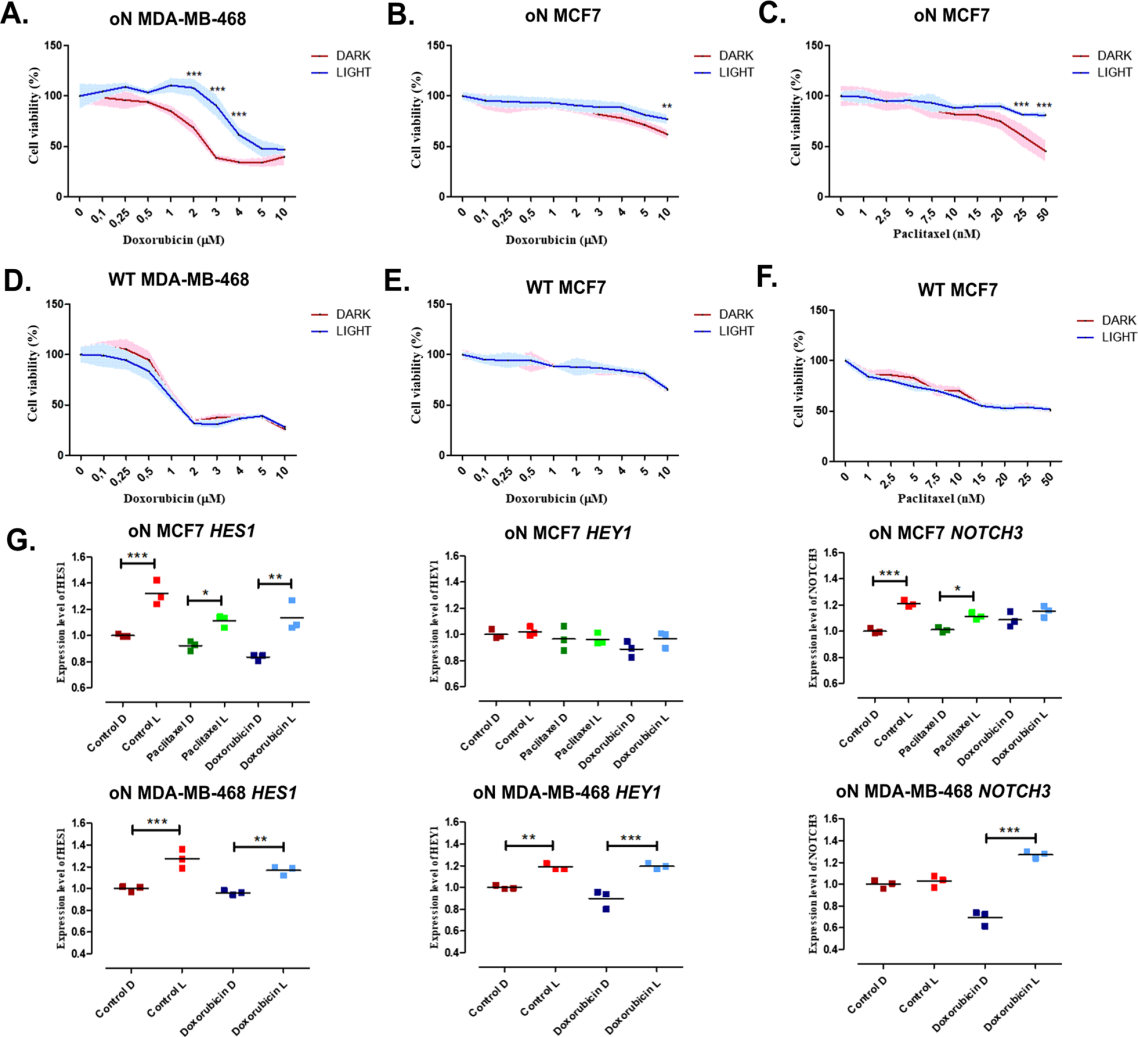
Cell chemoresistance induced by light activation of optoNotch (oN) in triple-negative (MDA-MB-468) and estrogen-positive (MCF7) breast cancer cell lines. Cells treated with various concentrations of doxorubicin or paclitaxel were activated by 0.05 s pulses of blue light for 3 h per day. After 96 h of drug treatment, viability of MCF7 and MDA-MB-468 cells was analyzed. OptoNotch-expressing cells were either photo-stimulated or kept in the dark, then exposed to different concentrations of anti-neoplastic agents (**a**–**c**). Wild type (WT) MCF7 and MDA-MB-468 were tested in parallel under identical conditions (**d**–**f**). The mRNA expression level for *HES1*, *HEY1* and *NOTCH3* assessed by qPCR 2^−∆∆^Ct method (normalization by *GAPDH*) in MCF7 and MDA-MB-468 cells after doxorubicin or paclitaxel treatment (**g**). The expression level of each gene was indicated as fold change compared to WT cells kept in the dark (Control D; indicated as 1). Control D—LOV2^V416L^-Zdk1-N1ICD stably expressing cells kept in the dark; Control L—light-activated LOV2^V416L^-Zdk1-N1ICD stably expressing cells; Paclitaxel\Doxorubicin D—LOV2^V416L^-Zdk1-N1ICD stably expressing cells kept in the dark treated with Paclitaxel\Doxorubicin, respectively. Paclitaxel\Doxorubicin L—light-activated LOV2^V416L^-Zdk1-N1ICD stable expressing cells treated with Paclitaxel\Doxorubicin, respectively. Data points represent each sample in triplicate and each graph is representative of three biological replicates. Statistical significance was analyzed by a one-way ANOVA test and Tukey’s multiple comparisons post-hoc test *vs*. not treated cells and it is presented as ***for p ≤ 0.001, **p ≤ 0.01; *p ≤ 0.05

The increased activity of NOTCH1 has been previously reported to induce drug resistance in various breast cancer lines [41–43]. To confirm that such resistance is the result of NOTCH1 activity, we tested the effect of oN activation on drug resistance in TNBC, MDA-MB-468, and estrogen-positive, MCF7, BC lines. These cells, WT or stably expressing optoNotch (oN), were then photo-stimulated or kept in the dark (controls). The next day, cells were exposed to different concentration of doxorubicin or paclitaxel. These drugs were selected based on the previous reports [41–43]. Light-activation of oN clearly induced doxorubicin resistance in MDA-MB-468 cells (Fig. 4a), while the same cells cultured in the dark, or WT cells photo-activated or not, showed no changes in drug sensitivity (Fig. 4d). Surprisingly, no statistically significant difference was observed between light-activated and non-activated oN MDA-MB-468 cells with paclitaxel treatment (Additional file 1: Fig. S4). In the case of the oN MCF7 cells, light stimulation resulted in greater chemoresistance to both doxorubicin (Fig. 4b) and paclitaxel (Fig. 4c). A trend that was not observed in stimulated or unstimulated WT MCF7 cells (Fig. 4e, f).

We then analyzed how the observed changes in chemoresistance by oN activation, relate to the expression of Notch1-target genes. The drug concentrations for this experiments were selected based on the viability tests described above (Fig. 3a–c). Thus, 3 µM doxorubicin was used on oN MDA-MB-468 and 10 µM doxorubicin and 50 µM paclitaxel was used on oN MCF7. As previously, the results showed that the *HES1* expression increased after light activation in both cell lines, regardless of the drug used. The expression level of *HEY1* increased after blue light activation in oN MDA-MB-468 but showed no changes in oN MCF7. Interestingly, a significant influence of drugs on *NOTCH3* expression was observed. In photo-activated oN MCF7, *NOTCH3* expression significantly increased, an effect that was nullified by doxorubicin treatment. The opposite effect was observed when light activated oN MDA-MB-468 cell were treated with doxorubicin, what resulted in an increase of *NOTCH3* expression (Fig. 4g).

Our results corroborate previous reports that suggested that high NOTCH1 activity fuels breast cancer cell proliferation [44] and chemoresistance [41–43, 45, 46]. Using optoNotch, we can discern that these effects are the result of NOTCH1 activity, since we eliminated variables such as simultaneous activation of several NOTCH receptors by ligands, both in cis- or trans- conformations, via gamma-secretase inhibition. The lack of chemoresistance to paclitaxel in TNBC cells is a surprising finding. Although, NOTCH1-induced paclitaxel resistance has only been reported in MCF7 cells [43], NOTCH signaling pathway dependent chemoresistance for another taxane has been reported for MDA-MB-231, another TNBC cell line [42], what suggest that NOTCH1 activity might promote drug resistance using different mechanisms depending of signaling and cellular context. Thus, this might be important to understand in detail for personalized medicine.

## Conclusions

OptoNotch allows the milli-second control of Notch1 signaling, both independently of cell–cell interactions or gamma-secretase activity. Here, we report that NOTCH1 activity is indeed involved in cell proliferation and drug resistance in breast cancers, both estrogen-depended and TNBC. Interestingly, cell type-specific responses were found in this study, which supports the concept that Notch signaling is context dependent, now we know not only in what ligand is presented but also what downstream genes are activated depending in other endogenous signaling pathways present in the cell.

oN could also be used in drug screening in search of N1ICD inhibitors or to estimate the role of this pathway in sensitization to other therapies e.g. in personalized medicine, as the differences we show in this work between TNBC and estrogen-dependent BC cells.

Using optoNotch we can specifically modulate N1ICD activation to discriminate between different modes of NOTCH1 activation, as suggested for the differential binding and force used by each of the ligands [47]. The fine-tuned activity of Notch receptors is likely the door to understand why these receptors often are found to have opposing downstream responses. Indeed, a similar system allowing spatiotemporal control of NOTCH1 activation was recently reported in *Drosophila* [48]. Light-controllable NOTCH pathway activation will find applications as a research tool to understand embryonic development, stemness and angiogenesis – processes where NOTCH1 plays crucial roles.

## Supplementary Information

**Additional file 1**. Nucleic acid sequence of the oN plasmid. Amino acid sequences of the engineered proteins. **Table S1.** Primers. **Fig. S1.** The MAGI-01 illumination instrument and the (LED) platform. **Fig. S2.** The mRNA expression of Notch1 downstream genes in oN breast cancer cell lines. **Fig. S3.** Representative images of the 3D cultures used for AMIDA analyses. **Fig. S4.** The influence of paclitaxel on the viability of oN MDA-MB-468 cells. Scripts used to process the data for AMIDA analysis.

## Data Availability

Supporting data can be found in the Additional file 1. All raw data is available on request from the corresponding author.

## Abbreviations

ADAM10: ADAM Metallopeptidase Domain 10
ADAM17: ADAM Metallopeptidase Domain 17
BC: Breast cancer
CSL: CBF1, suppressor of hairless, lag-1
D: Dark
DAPT: γ-Secretase inhibitor
DTX: Deltex E3 ubiquitin ligase
ECD: Extracellular domain
GSI: Gamma-secretase inhibitor
HEY: Hairy/enhancer-of-split related with YRPW motif
HES: Hairy/enhancer of split [E(spl)]) families of genes
JAG1: Jagged canonical notch ligand 1
L: Light
LOV2: Light-oxygen-voltage-sensing domain 2
Luc: Firefly luciferase
MAML: Mastermind like transcriptional coactivator 1
MTS: Myristoylation-targeting sequence
MYC: C-Myc protein
N1ICD: NOTCH1 intracellular domain
NICD: Notch intracellular domain
NRARP: NOTCH regulated ankyrin repeat protein
oN: OptoNotch
P2A: Porcine teschovirus-1 2A sequence
TMD: Transmembrane domain
TNBC: Triple-negative breast cancer
WT: Wild-type
Zdk1: Zdark-1 protein
2D: Two-dimensional
3D: Three-dimensional
