# Supplementary material for: Optogenetic control of NOTCH1 signaling
Source: Cell Commun Signal. 2022 May 18;20:67. doi: 10.1186/s12964-022-00885-5 (PMC9118860; doi:10.1186/s12964-022-00885-5)
Supplement: Supplementary file 2 — Additional file 1. Nucleic acid sequence of the oN plasmid. Amino acid sequences of the engineered proteins. Table S1. Primers. Fig. S1. The MAGI-01 illumination instrument and the (LED) platform. Fig. S2. The mRNA expression of Notch1 downstream genes in oN breast cancer cell lines. Fig. S3. Representative images of the 3D cultures used for AMIDA analyses. Fig. S4. The influence of paclitaxel on the viability of oN MDA-MB-468 cells. Scripts used to process the data for AMIDA analysis. [file 12964_2022_885_MOESM2_ESM.docx]

**Supplementary Material**

**Optogenetic control of NOTCH1 signaling**

Joanna Kałafut^1^, Jakub Czapiński^1^, Alicja Przybyszewska-Podstawka^1^,Arkadiusz Czerwonka^1^, Adrian Odrzywolski^1^, Cecilia Sahlgren^2,3^, Adolfo Rivero-Müller^1§^

^1^ Department of Biochemistry and Molecular Biology, Medical University of Lublin, 21-093, Lublin, Poland
^2^ Faculty of Science and Engineering, Biosciences, Åbo Akademi, Turku, Finland

^3^ Institute for Complex Molecular Systems, Eindhoven University of Technology, Eindhoven, the Netherlands

§ Correspondence: [a.rivero@umlub.pl](mailto:a.rivero@umlub.pl)

**Statements:**

All the raw data supporting the findings in this work can be obtained on request from the corresponding author.

Plasmids used in this work will be available via Addgene and on request from the corresponding author.

**Nucleic acid Sequence of the construct MTS-LOV2^WT^-P2A-Zdk-N1ICD (7142 bp)**

TAGTCTCAGATCTCGAGCTCAAGCTTCGAATTCTGCAGTCGACGGTACCGCGGGCCCGGGATCCACCGGATCTAGATAACTGATCATAATCAGCCATACCACATTTGTAGAGGTTTTACTTGCTTTAAAAAACCTCCCACACCTCCCCCTGAACCTGAAACATAAAATGAATGCAATTGTTGTTGTTAACTTGTTTATTGCAGCTTATAATGGTTACAAATAAAGCAATAGCATCACAAATTTCACAAATAAAGCATTTTTTTCACTGCATTCTAGTTGTGGTTTGTCCAAACTCATCAATGTATCTTAACGCGTAAATTGTAAGCGTTAATATTTTGTTAAAATTCGCGTTAAATTTTTGTTAAATCAGCTCATTTTTTAACCAATAGGCCGAAATCGGCAAAATCCCTTATAAATCAAAAGAATAGACCGAGATAGGGTTGAGTGTTGTTCCAGTTTGGAACAAGAGTCCACTATTAAAGAACGTGGACTCCAACGTCAAAGGGCGAAAAACCGTCTATCAGGGCGATGGCCCACTACGTGAACCATCACCCTAATCAAGTTTTTTGGGGTCGAGGTGCCGTAAAGCACTAAATCGGAACCCTAAAGGGAGCCCCCGATTTAGAGCTTGACGGGGAAAGCCGGCGAACGTGGCGAGAAAGGAAGGGAAGAAAGCGAAAGGAGCGGGCGCTAGGGCGCTGGCAAGTGTAGCGGTCACGCTGCGCGTAACCACCACACCCGCCGCGCTTAATGCGCCGCTACAGGGCGCGTCAGGTGGCACTTTTCGGGGAAATGTGCGCGGAACCCCTATTTGTTTATTTTTCTAAATACATTCAAATATGTATCCGCTCATGAGACAATAACCCTGATAAATGCTTCAATAATATTGAAAAAGGAAGAGTCCTGAGGCGGAAAGAACCAGCTGTGGAATGTGTGTCAGTTAGGGTGTGGAAAGTCCCCAGGCTCCCCAGCAGGCAGAAGTATGCAAAGCATGCATCTCAATTAGTCAGCAACCAGGTGTGGAAAGTCCCCAGGCTCCCCAGCAGGCAGAAGTATGCAAAGCATGCATCTCAATTAGTCAGCAACCATAGTCCCGCCCCTAACTCCGCCCATCCCGCCCCTAACTCCGCCCAGTTCCGCCCATTCTCCGCCCCATGGCTGACTAATTTTTTTTATTTATGCAGAGGCCGAGGCCGCCTCGGCCTCTGAGCTATTCCAGAAGTAGTGAGGAGGCTTTTTTGGAGGCCTAGGCTTTTGCAAAGATCGATCAAGAGACAGGATGAGGATCGTTTCGCATGATTGAACAAGATGGATTGCACGCAGGTTCTCCGGCCGCTTGGGTGGAGAGGCTATTCGGCTATGACTGGGCACAACAGACAATCGGCTGCTCTGATGCCGCCGTGTTCCGGCTGTCAGCGCAGGGGCGCCCGGTTCTTTTTGTCAAGACCGACCTGTCCGGTGCCCTGAATGAACTGCAAGACGAGGCAGCGCGGCTATCGTGGCTGGCCACGACGGGCGTTCCTTGCGCAGCTGTGCTCGACGTTGTCACTGAAGCGGGAAGGGACTGGCTGCTATTGGGCGAAGTGCCGGGGCAGGATCTCCTGTCATCTCACCTTGCTCCTGCCGAGAAAGTATCCATCATGGCTGATGCAATGCGGCGGCTGCATACGCTTGATCCGGCTACCTGCCCATTCGACCACCAAGCGAAACATCGCATCGAGCGAGCACGTACTCGGATGGAAGCCGGTCTTGTCGATCAGGATGATCTGGACGAAGAGCATCAGGGGCTCGCGCCAGCCGAACTGTTCGCCAGGCTCAAGGCGAGCATGCCCGACGGCGAGGATCTCGTCGTGACCCATGGCGATGCCTGCTTGCCGAATATCATGGTGGAAAATGGCCGCTTTTCTGGATTCATCGACTGTGGCCGGCTGGGTGTGGCGGACCGCTATCAGGACATAGCGTTGGCTACCCGTGATATTGCTGAAGAGCTTGGCGGCGAATGGGCTGACCGCTTCCTCGTGCTTTACGGTATCGCCGCTCCCGATTCGCAGCGCATCGCCTTCTATCGCCTTCTTGACGAGTTCTTCTGAGCGGGACTCTGGGGTTCGAAATGACCGACCAAGCGACGCCCAACCTGCCATCACGAGATTTCGATTCCACCGCCGCCTTCTATGAAAGGTTGGGCTTCGGAATCGTTTTCCGGGACGCCGGCTGGATGATCCTCCAGCGCGGGGATCTCATGCTGGAGTTCTTCGCCCACCCTAGGGGGAGGCTAACTGAAACACGGAAGGAGACAATACCGGAAGGAACCCGCGCTATGACGGCAATAAAAAGACAGAATAAAACGCACGGTGTTGGGTCGTTTGTTCATAAACGCGGGGTTCGGTCCCAGGGCTGGCACTCTGTCGATACCCCACCGAGACCCCATTGGGGCCAATACGCCCGCGTTTCTTCCTTTTCCCCACCCCACCCCCCAAGTTCGGGTGAAGGCCCAGGGCTCGCAGCCAACGTCGGGGCGGCAGGCCCTGCCATAGCCTCAGGTTACTCATATATACTTTAGATTGATTTAAAACTTCATTTTTAATTTAAAAGGATCTAGGTGAAGATCCTTTTTGATAATCTCATGACCAAAATCCCTTAACGTGAGTTTTCGTTCCACTGAGCGTCAGACCCCGTAGAAAAGATCAAAGGATCTTCTTGAGATCCTTTTTTTCTGCGCGTAATCTGCTGCTTGCAAACAAAAAAACCACCGCTACCAGCGGTGGTTTGTTTGCCGGATCAAGAGCTACCAACTCTTTTTCCGAAGGTAACTGGCTTCAGCAGAGCGCAGATACCAAATACTGTCCTTCTAGTGTAGCCGTAGTTAGGCCACCACTTCAAGAACTCTGTAGCACCGCCTACATACCTCGCTCTGCTAATCCTGTTACCAGTGGCTGCTGCCAGTGGCGATAAGTCGTGTCTTACCGGGTTGGACTCAAGACGATAGTTACCGGATAAGGCGCAGCGGTCGGGCTGAACGGGGGGTTCGTGCACACAGCCCAGCTTGGAGCGAACGACCTACACCGAACTGAGATACCTACAGCGTGAGCTATGAGAAAGCGCCACGCTTCCCGAAGGGAGAAAGGCGGACAGGTATCCGGTAAGCGGCAGGGTCGGAACAGGAGAGCGCACGAGGGAGCTTCCAGGGGGAAACGCCTGGTATCTTTATAGTCCTGTCGGGTTTCGCCACCTCTGACTTGAGCGTCGATTTTTGTGATGCTCGTCAGGGGGGCGGAGCCTATGGAAAAACGCCAGCAACGCGGCCTTTTTACGGTTCCTGGCCTTTTGCTGGCCTTTTGCTCACATGTTCTTTCCTGCGTTATCCCCTGATTCTGTGGATAACCGTATTACCGCCATGCATTAGTTATTAATAGTAATCAATTACGGGGTCAtTTAGTTCATAGCCCATATATGGAGTTCCGCGTTACATAACTTACGGTAAATGGCCCGCCTGGCTGACCGCCCAACGACCCCCGCCCATTGACGTCAATAATGACGTATGTTCCCATAGTAACGCCAATAGGGACTTTCCATTGACGTCAATGGGTGGAGTATTTACGGTAAACTGCCCACTTGGCAGTACATCAAGTGTATCATATGCCAAGTACGCCCCCTATTGACGTCAATGACGGTAAATGGCCCGCCTGGCATTATGCCCAGTACATGACCTTATGGGACTTTCCTACTTGGCAGTACATCTACGTATTAGTCATCGCTATTACCATGGTGATGCGGTTTTGGCAGTACATCAATGGGCGTGGATAGCGGTTTGACTCACGGGGATTTCCAAGTCTCCACCCCATTGACGTCAATGGGAGTTTGTTTTGGCACCAAAATCAACGGGACTTTCCAAAATGTCGTAACAACTCCGCCCCATTGACGCAAATGGGCGGTAGGCGTGTACGGTGGGAGGTCTATATAAGCAGAGCTGGTTTAGTGAACCGTCAGATCCGCTAGCGCTACCGGTCGCCACCatgGGCTGCGTGCAGTGCAAGGACAAGGAGGCCACCAAGCTGACCGAGGAGTTGGCTACTACACTTGAACGTATTGAGAAGAACTTTGTCATTACTGACCCAAGATTGCCAGATAATCCCATTATATTCGCGTCCGATAGTTTCTTGCAGTTGACAGAATATAGCCGTGAAGAAATTTTGGGAAGAAACTGCAGGTTTCTACAAGGTCCTGAAACTGATCGCGCGACAGTGAGAAAAATTAGAGATGCCATAGATAACCAAACAGAGGTCACTGTTCAGCTGATTAATTATACAAAGAGTGGTAAAAAGTTCTGGAACCTCTTTCACTTGCAGCCTATGCGAGATCAGAAGGGAGATGTCCAGTACTTTATTGGGGTTCAGTTGGATGGAACTGAGCATGTCCGAGATGCTGCCGAGAGAGAGGGAGTCATGCTGATTAAGAAAACTGCAGAAAATATTGATGAGGCGGCAAAAGAACTTGGATCAGGAGCTACAAATTTTTCACTCCTCAAGCAAGCAGGAGACGTGGAAGAGAATCCCGGTCCTatggtggataacaaattcaataaagaaaagacgcgtgccggtgcggaaatccattctctgccaaacctgaatgttgagcagaagtttgccttcatcgtgagcctgtttgatgatccatctcagagcgccaatctgctggccgaagccaaaaaactgaacgatgcccaggccccaaaaggtggttctggtggtaGCCGGCGGCAGCATGGCCAGCTCTGGTTCCCTGAGGGCTTCAAAGTGTCTGAGGCCAGCAAGAAGAAGCGGCGGGAGCCCCTCGGCGAGGACTCCGTGGGCCTCAAGCCCCTGAAGAACGCTTCAGACGGTGCCCTCATGGACGACAACCAGAATGAGTGGGGGGACGAGGACCTGGAGACCAAGAAGTTCCGGTTCGAGGAGCCCGTGGTTCTGCCTGACCTGGACGACCAGACAGACCACCGGCAGTGGACTCAGCAGCACCTGGATGCCGCTGACCTGCGCATGTCTGCCATGGCCCCCACACCGCCCCAGGGTGAGGTTGACGCCGACTGCATGGACGTCAATGTCCGCGGGCCTGATGGCTTCACCCCGCTCATGATCGCCTCCTGCAGCGGGGGCGGCCTGGAGACGGGCAACAGCGAGGAAGAGGAGGACGCGCCGGCCGTCATCTCCGACTTCATCTACCAGGGCGCCAGCCTGCACAACCAGACAGACCGCACGGGCGAGACCGCCTTGCACCTGGCCGCCCGCTACTCACGCTCTGATGCCGCCAAGCGCCTGCTGGAGGCCAGCGCAGATGCCAACATCCAGGACAACATGGGCCGCACCCCGCTGCATGCGGCTGTGTCTGCCGACGCACAAGGTGTCTTCCAGATCCTGATCCGGAACCGAGCCACAGACCTGGATGCCCGCATGCATGATGGCACGACGCCACTGATCCTGGCTGCCCGCCTGGCCGTGGAGGGCATGCTGGAGGACCTCATCAACTCACACGCCGACGTCAACGCCGTAGATGACCTGGGCAAGTCCGCCCTGCACTGGGCCGCCGCCGTGAACAATGTGGATGCCGCAGTTGTGCTCCTGAAGAACGGGGCTAACAAAGATATGCAGAACAACAGGGAGGAGACACCCCTGTTTCTGGCCGCCCGGGAGGGCAGCTACGAGACCGCCAAGGTGCTGCTGGACCACTTTGCCAACCGGGACATCACGGATCATATGGACCGCCTGCCGCGCGACATCGCACAGGAGCGCATGCATCACGACATCGTGAGGCTGCTGGACGAGTACAACCTGGTGCGCAGCCCGCAGCTGCACGGAGCCCCGCTGGGGGGCACGCCCACCCTGTCGCCCCCGCTCTGCTCGCCCAACGGCTACCTGGGCAGCCTCAAGCCCGGCGTGCAGGGCAAGAAGGTCCGCAAGCCCAGCAGCAAAGGCCTGGCCTGTGGAAGCAAGGAGGCCAAGGACCTCAAGGCACGGAGGAAGAAGTCCCAGGACGGCAAGGGCTGCCTGCTGGACAGCTCCGGCATGCTCTCGCCCGTGGACTCCCTGGAGTCACCCCATGGCTACCTGTCAGACGTGGCCTCGCCGCCACTGCTGCCCTCCCCGTTCCAGCAGTCTCCGTCCGTGCCCCTCAACCACCTGCCTGGGATGCCCGACACCCACCTGGGCATCGGGCACCTGAACGTGGCGGCCAAGCCCGAGATGGCGGCGCTGGGTGGGGGCGGCCGGCTGGCCTTTGAGACTGGCCCACCTCGTCTCTCCCACCTGCCTGTGGCCTCTGGCACCAGCACCGTCCTGGGCTCCAGCAGCGGAGGGGCCCTGAATTTCACTGTGGGCGGGTCCACCAGTTTGAATGGTCAATGCGAGTGGCTGTCCCGGCTGCAGAGCGGCATGGTGCCGAACCAATACAACCCTCTGCGGGGGAGTGTGGCACCAGGCCCCCTGAGCACACAGGCCCCCTCCCTGCAGCATGGCATGGTAGGCCCGCTGCACAGTAGCCTTGCTGCCAGCGCCCTGTCCCAGATGATGAGCTACCAGGGCCTGCCCAGCACCCGGCTGGCCACCCAGCCTCACCTGGTGCAGACCCAGCAGGTGCAGCCACAAAACTTACAGATGCAGCAGCAGAACCTGCAGCCAGCAAACATCCAGCAGCAGCAAAGCCTGCAGCCGCCACCACCACCACCACAGCCGCACCTTGGCGTGAGCTCAGCAGCCAGCGGCCACCTGGGCCGGAGCTTCCTGAGTGGAGAGCCGAGCCAGGCAGACGTGCAGCCACTGGGCCCCAGCAGCCTGGCGGTGCACACTATTCTGCCCCAGGAGAGCCCCGCCCTGCCCACGTCGCTGCCATCCTCGCTGGTCCCACCCGTGACCGCAGCCCAGTTCCTGACGCCCCCCTCGCAGCACAGCTACTCCTCGCCTGTGGACAACACCCCCAGCCACCAGCTACAGGTGCCTGAGCACCCCTTCCTCACCCCGTCCCCTGAGTCCCCTGACCAGTGGTCCAGCTCGTCCCCGCATTCCAACGTCTCCGACTGGTCCGAGGGCGTCTCCAGCCCTCCCACCAGCATGCAGTCCCAGATCGCCCGCATCCCGGAGGCGTTCAAGTAA

**Nucleic acid Sequence of the LOV2^V416L^**

TTGGCTACTACACTTGAACGTATTGAGAAGAACTTTCTCATTACTGACCCAAGATTGCCAGATAATCCCATTATATTCGCGTCCGATAGTTTCTTGCAGTTGACAGAATATAGCCGTGAAGAAATTTTGGGAAGAAACTGCAGGTTTCTACAAGGTCCTGAAACTGATCGCGCGACAGTGAGAAAAATTAGAGATGCCATAGATAACCAAACAGAGGTCACTGTTCAGCTGATTAATTATACAAAGAGTGGTAAAAAGTTCTGGAACCTCTTTCACTTGCAGCCTATGCGAGATCAGAAGGGAGATGTCCAGTACTTTATTGGGGTTCAGTTGGATGGAACTGAGCATGTCCGAGATGCTGCCGAGAGAGAGGGAGTCATGCTGATTAAGAAAACTGCAGAAAATATTGATGAGGCGGCAAAAGAACTT

**Amino acid Sequence of the chimeric proteins:**

**MTS-LOV2^WT^ (160 aa)**

MGCVQCKDKEATKLTEELATTLERIEKNFVITDPRLPDNPIIFASDSFLQLTEYSREEILGRNCRFLQGPETDRATVRKIRDAIDNQTEVTVQLINYTKSGKKFWNLFHLQPMRDQKGDVQYFIGVQLDGTEHVRDAAEREGVMLIKKTAENIDEAAKEL

**MTS-LOV2^V416L^ (160 aa)**

MGCVQCKDKEATKLTEELATTLERIEKNFLITDPRLPDNPIIFASDSFLQLTEYSREEILGRNCRFLQGPETDRATVRKIRDAIDNQTEVTVQLINYTKSGKKFWNLFHLQPMRDQKGDVQYFIGVQLDGTEHVRDAAEREGVMLIKKTAENIDEAAKEL

**Zdk1-flex-N1ICD (860 aa)**

MVDNKFNKEKTRAGAEIHSLPNLNVEQKFAFIVSLFDDPSQSANLLAEAKKLNDAQAPKGGSGGSRRQHGQLWFPEGFKVSEASKKKRREPLGEDSVGLKPLKNASDGALMDDNQNEWGDEDLETKKFRFEEPVVLPDLDDQTDHRQWTQQHLDAADLRMSAMAPTPPQGEVDADCMDVNVRGPDGFTPLMIASCSGGGLETGNSEEEEDAPAVISDFIYQGASLHNQTDRTGETALHLAARYSRSDAAKRLLEASADANIQDNMGRTPLHAAVSADAQGVFQILIRNRATDLDARMHDGTTPLILAARLAVEGMLEDLINSHADVNAVDDLGKSALHWAAAVNNVDAAVVLLKNGANKDMQNNREETPLFLAAREGSYETAKVLLDHFANRDITDHMDRLPRDIAQERMHHDIVRLLDEYNLVRSPQLHGAPLGGTPTLSPPLCSPNGYLGSLKPGVQGKKVRKPSSKGLACGSKEAKDLKARRKKSQDGKGCLLDSSGMLSPVDSLESPHGYLSDVASPPLLPSPFQQSPSVPLNHLPGMPDTHLGIGHLNVAAKPEMAALGGGGRLAFETGPPRLSHLPVASGTSTVLGSSSGGALNFTVGGSTSLNGQCEWLSRLQSGMVPNQYNPLRGSVAPGPLSTQAPSLQHGMVGPLHSSLAASALSQMMSYQGLPSTRLATQPHLVQTQQVQPQNLQMQQQNLQPANIQQQQSLQPPPPPPQPHLGVSSAASGHLGRSFLSGEPSQADVQPLGPSSLAVHTILPQESPALPTSLPSSLVPPVTAAQFLTPPSQHSYSSPVDNTPSHQLQVPEHPFLTPSPESPDQWSSSSPHSNVSDWSEGVSSPPTSMQSQIARIPEAFK

**Table 1.** List of primers.

|  | **Forward** | **Reverse** |
| --- | --- | --- |
| LOV2 mutagenesis (LOV2^V416L^) | 5’-GAACTTTCTCATTACTGACCCAAGATTGCC-3’ | 5’-GGTCAGTAATGAGAAAGTTCTTCTCAATACGTTC-3’ |
| *GAPDH* | 5′-CTCTGCTCCTCCTGTTCGAC-3’ | 5′- GCCCAATACGACCAAATCC-3’ |
| *HES1* | 5′- TCAACACGACACCGGATAAAC-3’ | 5′- GCCGCGAGCTATCTTTCTTCA-3’ |
| *HEY1* | 5′- CGGCTCTAGGTTCCATGTCC-3’ | 5’- GCTTAGCAGATCCCTGCTTCT-3’ |
| *NOTCH3* | 5′- GCAGATGGCTCAACGGCACTG-3’ | 5’-GGGGTCTCCTCCTTGCTATCCTG-3’ |


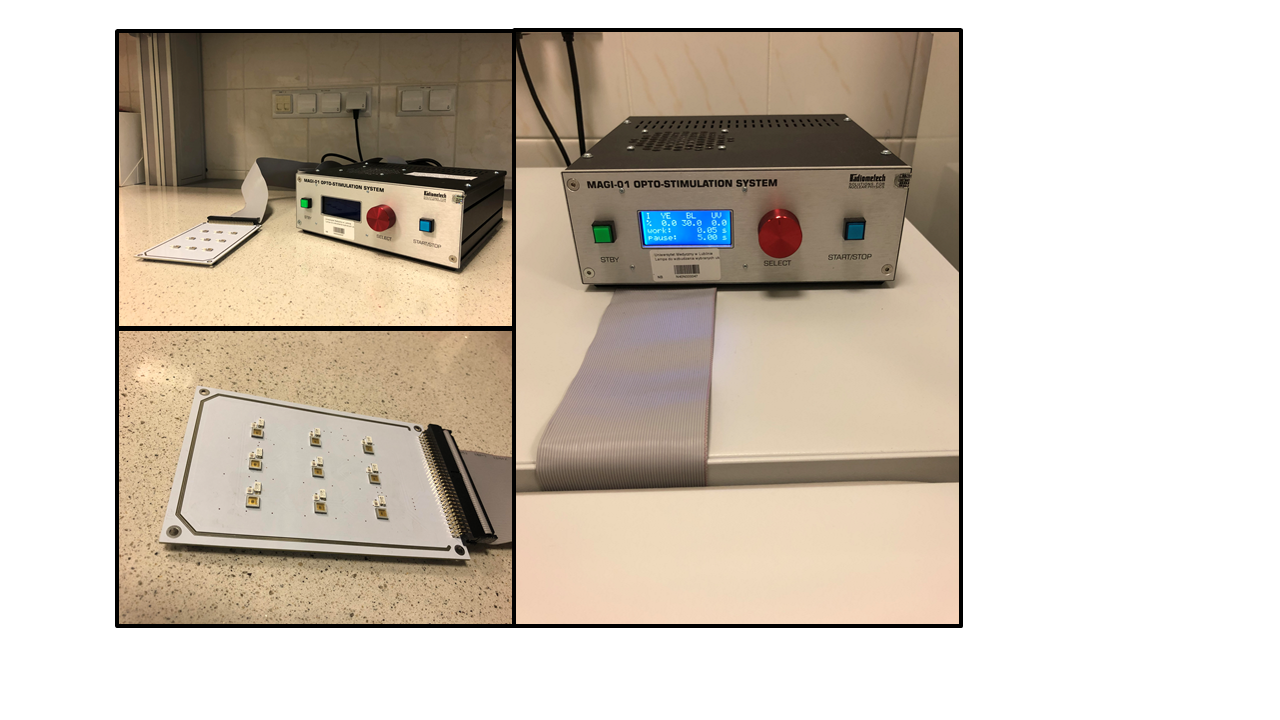


**Suppl. Fig. 1.** The MAGI-01 machine and the illumination (LED) platform. The control panel and the diode illumination platform (left), which has been designed to fit in a cell culture incubator (right image).


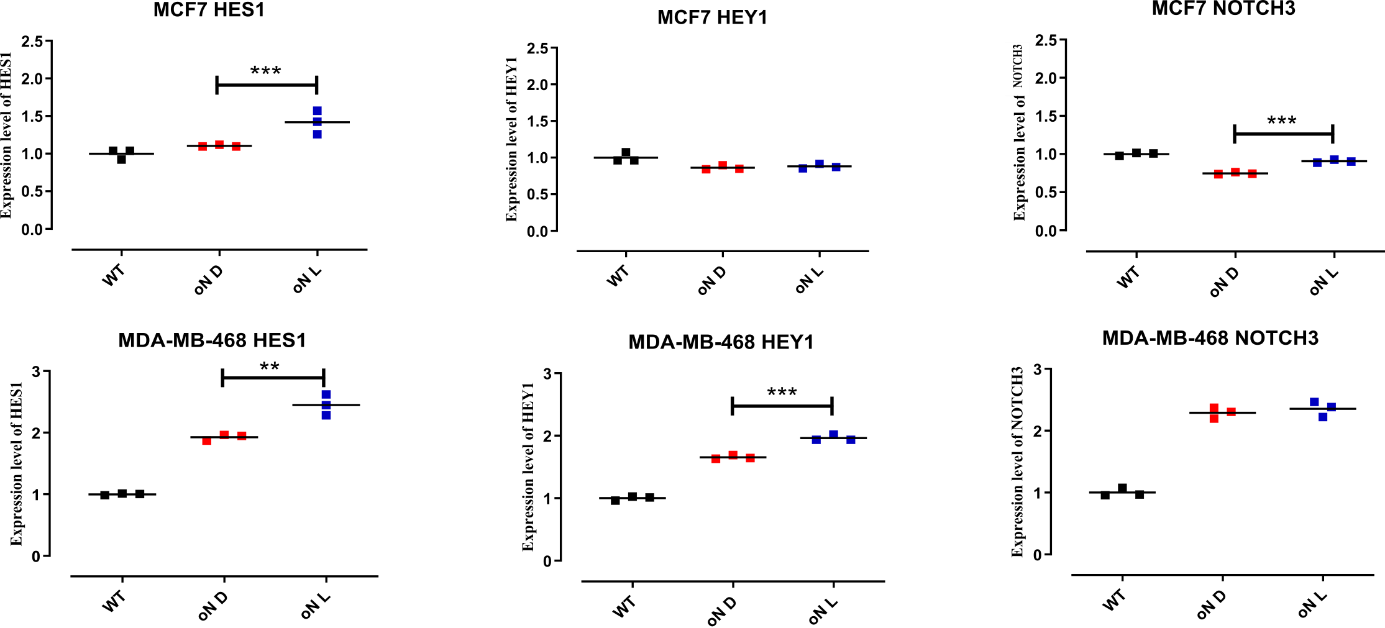


**Suppl. Fig. 2.** The mRNA expression level for *HES1*, *HEY1* and *NOTCH3* assessed by qPCR 2-∆∆Ct (normalization by *GAPDH*) in oN MCF7 and oN MDA-MB-468 cells. WT (wild-type cells) is indicated as 1. All data were analyzed with a one-way ANOVA test and Tukey’s multiple comparisons post-hoc test *vs*. not light stimulated cells (D; dark). *, p≤0.05; **, p≤0.01; ***, p≤0.001 were considered statistically significant.


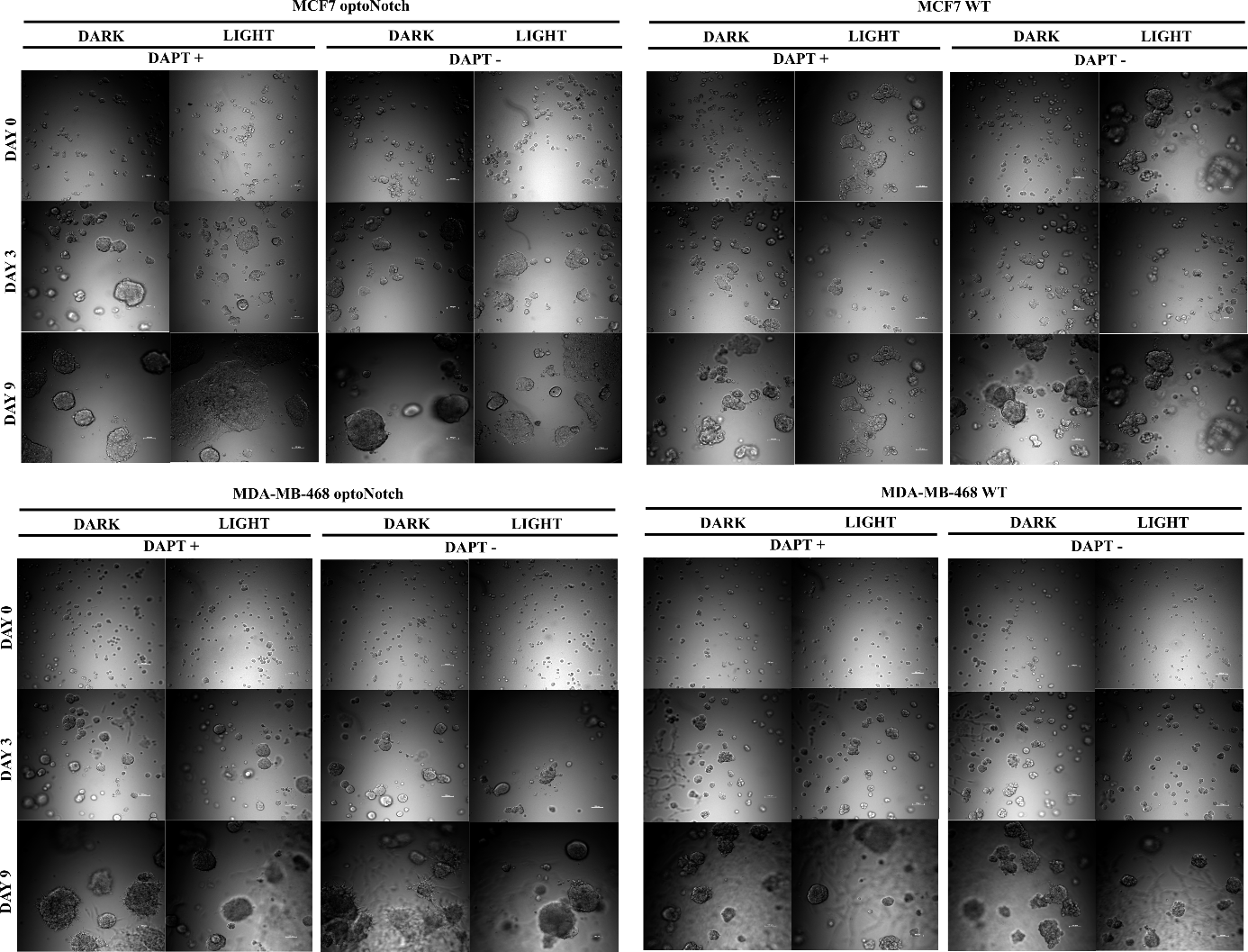


**Suppl. Fig. 3.** Representative images of oN and WT MCF7 and MDA-MB-468 cell spheroids cultured with or without DAPT after light activation, compared to those cells without light activation. Images acquired by confocal microscopy.


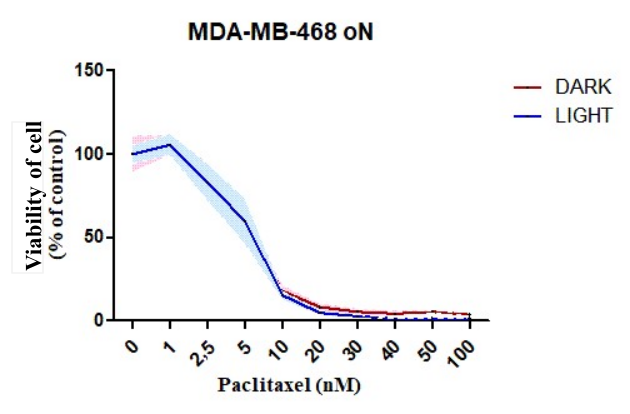


**Suppl. Fig. 4.** OptoNotch-expressing MDA-MB-468 (oN MDA-MB-468) cells photo-stimulated or kept in the dark, then exposed to different concentrations of Paclitaxel. After 96 hours of drug treatment, cells was analysed in MTT test. Data points represent each sample in triplicate and each graph is representative of three biological replicates.
